# Breath tests for gastrointestinal diseases - will it be safe to conduct breath tests after the COVID-19 pandemic?

**DOI:** 10.6061/clinics/2020/e2092

**Published:** 2020-06-16

**Authors:** Rejane Mattar

**Affiliations:** Divisao de Gastroenterologia e Hepatologia Clinica, Hospital das Clinicas HCFMUSP, Faculdade de Medicina, Universidade de Sao Paulo, Sao Paulo, SP, BR

Breath tests are simple, noninvasive alternatives, which are considered harmless for the diagnosis of *Helicobacter pylori* (*H. pylori*) infection, small intestinal bacterial overgrowth (SIBO), lactose intolerance (1), and inflammatory bowel disease (2). In Brazil, the acquisition of ^13^C-urea from abroad is challenging; however, with the support of the Division of Pharmacy, ^13^C-urea breath tests were routinely performed in clinical practice (3). Patients using anticoagulant drugs, with low platelets count, cardiac impairment that contraindicates endoscopy, or those who did not want to undergo upper gastrointestinal endoscopy had the benefit of undergoing ^13^C-urea breath tests for *H. pylori* infection diagnosis, instead.

These scenarios changed abruptly by the middle of March this year, when the cases of coronavirus disease 2019 (COVID-19), caused by the severe acute respiratory syndrome coronavirus-2 (SARS-CoV-2) (4) started to rise in Brazil. According to WHO (https://covid19.who.int/), at the beginning of June, there were 514,849 confirmed cases and 29,314 deaths in Brazil. The performance of breath tests usually considered risk-free, are currently threatened by SARS-CoV-2 (5).

The symptoms of COVID-19 most commonly were fever, cough and expectoration, sore throat, headache, fatigue, and shortness of breath (6); conversely, some patients with mild disease presented digestive symptoms (diarrhea, nausea, and vomiting) with or without respiratory symptoms (7). Diarrhea was the first symptom with or without fever, that lasted from 1-14 days. These patients with digestive symptoms had longer a duration between disease onset and viral clearance, with positive RNA virus in the feces, than those with respiratory symptoms (7).

Patients with lactose intolerance, irritable bowel syndrome, and SIBO usually have diarrhea (1); thus, caution must be taken, as patients with only diarrhea may be harboring SARS-CoV-2, and may unknowingly spread the virus (7) in clinical investigations using H_2_ breath tests to detect lactose intolerance or SIBO (1). To exclude lactose intolerance, a genetic test is a safer choice than the lactose breath test (8).

The instruments to measure H_2_, generated by bacterial fermentation of carbohydrates, in end-alveolar breath samples, may be stationary (CM2 microlyzer, Breath tracker analyzer) or portable (Gastrolyzer, Easy H_2_). When using stationary equipment, the patient blows into a plastic device, consisting of a mouthpiece and T valve attached to a plastic bag. The breath is aspirated into a 30 mL syringe attached to the T valve (8). When using portable equipment, the patient inspires the air deeply, holds the air inside the lung for 15 seconds, and then blows all the air into a mouthpiece attached to the equipment. The patient forces expiration that generates respiratory droplets and aerosols. Viral RNA was detected in respiratory droplets and aerosols from coronavirus-, influenza virus-, and rhinovirus-infected patients (9). The patient blows the H_2_ baseline collection and several times after swallowing a carbohydrate substrate. The exam lasts 1 hour and 20 minutes for SIBO and three hours for lactose intolerance. The test carries risks of contamination to the healthcare worker collecting the breath samples and to the patients, as the ultrafine aerosol droplets may also carry SARS-CoV-2 and remain airborne for long periods, and could be inhaled (5).

For the ^13^C-urea breath test for *H. pylori* infection diagnosis, the patient blows into a plastic mouthpiece attached to an aluminized bag. Patients inspire the air and blow with strain until the bag is completely full of air. Sometimes it stimulates cough. Breath samples are collected before and after ingesting a ^13^C-urea capsule (3). Although the test lasts 20 minutes, it carries the risk of contamination by SARS-CoV-2 in the aerosol droplets generated by the exhaled air (5).

One possibility to overcome the risks of contamination is a pre-procedural mouth rinse, including chlorhexidine, cetylpyridinium chloride, and essential oils that decreases the proportion of microorganisms in oral aerosols, and leads to a mean reduction of 68.4% colony-forming units in dental aerosols. The impact on coronavirus is uncertain; however, it is efficient against human immunodeficiency, herpes simplex, and hepatitis B viruses (10). Wearing protective glasses, face shields, surgical masks, or particulate respirator Health-certified N95, or equivalent (10), may protect the healthcare worker, but not the patient that has to blow inside a room with oral aerosols, generated by previous exams. Patient selection should be done based on a negative swab for SARS-CoV-2 by real-time reverse transcription-PCR; however, the nasopharyngeal swab specimen has to be properly collected by inserting deeply into the nasal cavity, or it can produce a false negative (11). Other analytical assays for SARS-CoV-2 diagnosis include antigen detection by lateral flow assays that are fast and low cost; nonetheless, these tests lack good sensitivity early in the infection stage (11). Serology detection, an indirect method, is reliable to indicate past infection than active ones (11). It would be better to wait for a vaccine before resuming breath tests again, but it is unknown how long that will take.
